# Tunable magnetic states on the zigzag edges of hydrogenated and halogenated group-IV nanoribbons

**DOI:** 10.1038/srep39083

**Published:** 2016-12-16

**Authors:** Tzu-Cheng Wang, Chia-Hsiu Hsu, Zhi-Quan Huang, Feng-Chuan Chuang, Wan-Sheng Su, Guang-Yu Guo

**Affiliations:** 1 Department of Physics, National Sun Yat-Sen University, Kaohsiung 80424, Taiwan; 2 Department of Physics and Center for Theoretical Sciences, National Taiwan University, Taipei 10617, Taiwan; 3 Experimentation Division, National Taiwan Science Education Center, Taipei 11165, Taiwan; 4 Department of Electro-Optical Engineering, National Taipei University of Technology, Taipei 10608, Taiwan; 5 Physics Division, National Center for Theoretical Sciences, Hsinchu 30013, Taiwan

## Abstract

The magnetic and electronic properties of hydrogenated and halogenated group-IV zigzag nanoribbons (ZNRs) are investigated by first-principles density functional calculations. Fascinatingly, we find that all the ZNRs have magnetic edges with a rich variety of electronic and magnetic properties tunable by selecting the parent and passivating elements as well as controlling the magnetization direction and external strain. In particular, the electric property of the edge band structure can be tuned from the conducting to insulating with a band gap up to 0.7 eV. The last controllability would allow us to develop magnetic on-off nano-switches. Furthermore, ZNRs such as SiI, Ge, GeI and SnH, have fully spin-polarized metallic edge states and thus are promising materials for spintronics. The calculated magnetocrystalline anisotropy energy can be as large as ~9 meV/edge-site, being 2×10^3^ time greater than that of bulk Ni and Fe (~5 μeV/atom), and thus has great potential for high density magneto-electric data-storage devices. Finally, the calculated exchange coupling strength and thus magnetic transition temperature increases as the applied strain goes from −5% to 5%. Our findings thus show that these ZNRs would have exciting applications in next-generation electronic and spintronic nano-devices.

Two-dimensional (2D) materials have recently received phenomenal interest since graphene, the archetypal 2D material, was discovered in 2004. Graphene, a single-atom-thick honeycomb lattice sheet of sp^2^-bonded carbon atoms with a zero bandgap due to its conduction and valence bands meeting at the Dirac points, has been extensively studied because of its unusual electrical, optical and mechanical properties^1^. Nevertheless, the semi-metallic property of graphene considerably limits its technological applications. Therefore, much effort has been made to create a band gap in graphene in order to extend its functions and applications. On the other hand, inspired by the current graphene research, an increasing number of researchers have turned their attention to exploring other 2D materials, such as monolayers of boron nitride and MoS_2_^2^,^3^,^4^. These graphene-like 2D materials exhibit notably different properties, not only from graphene but also from each other. They have a wide range of potential applications, including nanoelectronics, nanophotonics^5^, catalysts^6^, energy related systems^7^, and biomedicine^8^.

Of particular interest here is the fact that graphene, with band gap openings at the Dirac points by the spin-orbit coupling (SOC), is also the first topological insulator proposed to host the quantum spin Hall effect^9^,^10^. Therefore, because of its dissipationless edge spin current transport, graphene as a quantum spin Hall insulator further promises exciting applications in spintronics. However, the SOC band gaps in graphene are too small (~0.01 meV) to be useful for any real applications. It is thus natural to study if graphene-like systems may be formed by other group-IV elements such as Si, Ge and Sn, which have a much larger spin-orbit interaction. Like graphene, all the group-IV honeycomb crystals are zero-gap semiconductors if the SOC is ignored. However, much stronger SOC in these group-IV honeycomb crystals opens a significant topologically nontrivial bandgap and turns these crystals into 2D topological insulators^11^,^12^,^13^,^14^,^15^. Different from graphene, the basal planes of these group-IV graphene-like materials (known as silicene, germanene and stanene), are buckled with a chair-like buckled conformation rather than completely flat. Due to the stronger SOC, lower spatial symmetry, and different chemistry as compared to graphene, these group-IV graphene-like 2D materials exhibit many new interesting features.

As a truly 2D system, graphene shows many intriguing physical properties. However, to build device structures with desired functionalities, the infinite graphitic sheet must be cut into pieces of specific dimensions (e.g., nanoribbons) suitable for technological applications. Graphene nanoribbons (GNRs), which are one-dimensional (1D) nanostructures, are fabricated in laboratories by cutting them out from infinite 2D graphene sheet along a given direction. There are two primary ways to cut out the nanoribbons, and the two resultant structures are known as armchair and zigzag nanoribbons, respectively. The zigzag GNRs (ZGNRs) possess flat edge states with a singular density of states (DOS) and are thus susceptible to the magnetic instability. Indeed, the ZGNRs have been predicted to half-metallic, ferromagnetic, and antiferromagnetic with considerable magnetic moments located at the edge sites^16^,^17^,^18^. In contrast, the armchair GNRs are semiconductors with an energy gap which decreases with the increasing nanoribbon width^17^,^18^, and thus have no magnetism. Similar to graphene, other group-IV^19^,^20^ and group-V^21^ based sheets can be shaped to nanoribbons also with either armchair or zigzag edges, which dramatically influence the physical properties especially electricity and magnetism, of the resultant nanoribbon materials. For instance, the ground state of pristine zigzag phosphorene nanoribbons (ZPNRs)^21^ prefers ferromagnetic state in the same edge but antiferromagnetic state between two opposite edges. Such a magnetization originates from the instability induced by the half-filled one-dimensional bands crossing the Fermi level, while for ZGNRs the magnetization arises from the peculiar edge localized states near the Fermi level.

In this work, we carry out a systematic study of the magnetic and electronic properties of the zigzag hydrogenated and halogenated group-IV (Si, Ge and Sn) nanoribbons without and with an applied strain, by performing extensive first-principles density functional calculations. We find that all the zigzag nanoribbons have magnetic edges with a rich variety of electronic and magnetic properties, depending on the parent and passivating elements as well as the magnetization direction and external strain. Thus, these zigzag nanoribbons would have exciting applications in next-generation electronic and spintronic nano-devices.

## Results

Similar to graphene, the structural and electronic properties of the pristine group-IV honeycomb monolayers (i.e., silicene, germanene and stanene) can be altered by surface hydrogenation and halogenations^11^,^12^,^13^,^14^,^15^,^22^. The computed Z_2_ numbers^23^ of the functionalized group IV honeycomb monolayers are summarized in Table 1. The electronic structures of the hydrogenated group IV monolayers are found to be topologically trivial. However, the electronic structures of halogenated Ge and Sn monolayers are found to be topologically nontrivial, although that of halogenated Si monolayers are topologically trivial. The topological phase transition from the trivial halogenated Si monolayers to non-trivial halogenated Ge and Sn monolayers was attributed to the enlarged lattice constants in the latter cases^11^,^12^,^13^,^14^,^15^,^22^.

**Figure 1:**
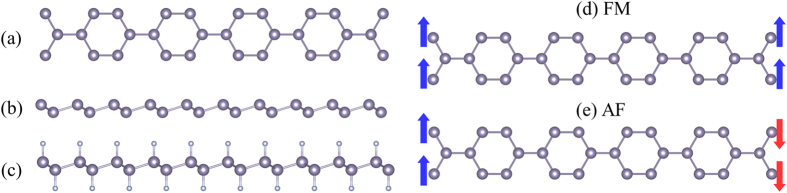
(**a**) The top view of the honeycomb zigzag nanoribbon (ZNR) structure. Side view of (**b**) prime and (**c**) adsorbed ZNRs. (**d**) Ferromagnetic (FM) and (**e**) antiferromagnetic (AF) configurations at the nanoribbon edge. Blue and red arrows represent up and down spins, respectively.

As listed in Table 2, the lattice constants of theoretically optimized pristine and functionalized group IV monolayers are in good agreement with previous calculations^11^,^12^,^13^,^14^,^15^,^22^. We construct the 1D nanoribbons by cutting them out from the corresponding 2D sheets along the zigzag direction. Such a zigzag nanoribbon (ZNR) can be indexed by its width *N*, the number of zigzag C-C chains across the nanoribbon. Figure 1(a) illustrates such a ZNR with 10 zigzag C-C chains, denoted as 10-ZNR, which is built as the representative ZNR to characterize the edge structure and electronic properties of the ZNRs. Figure 1 (b) and (c) are the side-views of the prime and functionalized ZNRs, respectively. To explore possible magnetism in the considered ZNRs, we considered two spin configurations, namely, ferromagnetic (FM) and antiferromagnetic (AF), as depicted in Fig. 1 (d) and (e). The calculated total energy difference between the nonmagnetic (NM) and FM configurations as well as between the FM and AF configurations, and the corresponding magnetic moments are summarized in Table 2. Interestingly, in all the studied ZNRs, magnetic states are favored over the NM state. Table 2 shows that the energy of a magnetic configuration (either FM or AF) can be lower than that of the NM one by as much as several hundreds of meV. As expected, the energy difference between the FM and AF configurations is much smaller (being ~1.0 meV) (Table 2), indicating the magnetic coupling between the two edges of each ZNR being weak.

To determine the exchange coupling *J* between two neighboring atoms along the same edge of a ZNR, we also calculate the total energy of both the intra-edge FM and AF configurations. We then estimate the *J* value by mapping the calculated total energies to the Heisenberg model *H* = *−*∑_*<i,j>*_*J*_*ij*_*σ*_*i*_*·σ*_*j*_, where *σ*_*i*_ is the unit vector denoting the direction of the spin at site *I* and the notation <*i,j*> indicates that only nearest neighbor pairs are considered in the sum. The obtained exchange parameters *J* are listed in Table 2. We find that for all the Ge and Sn ZNRs except GeH, the intra-edge FM coupling is favored, while, in contrast, an intra-edge AF coupling is preferred in all the Si ZNRs (Table 2). Interestingly, this suggests that one can choose either a FM or AF spin state at will by simply choosing a different group IV element. Furthermore, we use the obtained *J* values to estimate the Curie (Neel) temperature within the mean field approximation as *k*_*B*_*T*_*C*_  = (1/3)*zJ*, where the coordination number *z* equals to 2 and *k*_*B*_ is the Boltzmann constant^24^. The thus-estimated *T*_*C*_ (*T*_*N*_) values are listed in Table 2. Remarkably, the estimated *T*_*C*_ for the fluorinated zigzag stanene nanoribbon is as high as ~140 K, which is higher than the temperature of liquid nitrogen.

**Figure 2:**
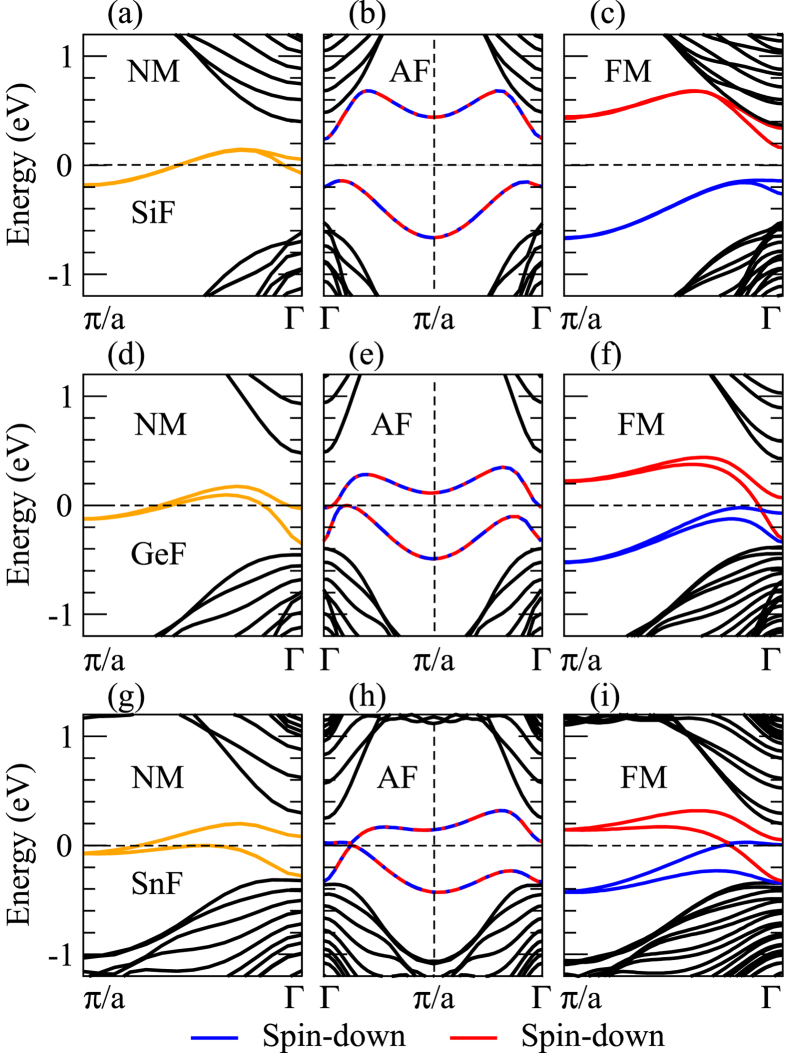
The band structures of (**a**–**c**) SiF, (**d**–**f**) GeF and (**g**–**i**) SnF zigzag nanoribbons (ZNRs) in the NM, FM and AF states, respectively. The band structures were calculated with the SOC included and magnetization being off-plane. The colored curves represent the contributions from the edge atoms on the ZNRs. The blue and red curves represent the spin-up and spin-down states, respectively.

**Figure 3:**
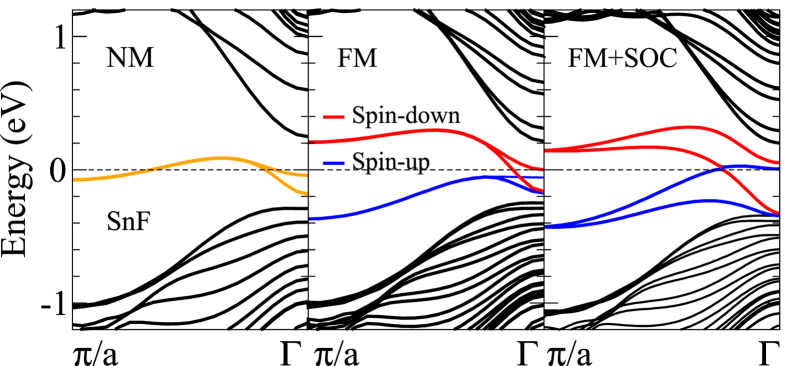
The band structures of the SnF zigzag nanoribbon in the (left) nonmagnetic (NM), (middle) ferromagnetic (FM) and (right) FM plus spin-orbit coupling (SOC) states. In the FM and FM+SOC cases, the magnetization is off-plane. The colored curves represent the contributions from the edge atoms on the ZNR. The blue and red curves represent the spin-up and spin-down states, respectively.

To analyze the electronic and magnetic properties of the studied ZNRs, we calculate their band structures in the NM, FM and AF states with the SOC included. Since the hydrogenated and halogenated 2D materials exhibit similar band structures, we show only the band structures for the fluorinated silicene, germanene and stanene ZNRs as representatives in Fig. 2. It is found that the electronic structures of the ZNRs can be significantly modified by chemical adsorption, as has been reported in previous investigations^11^,^12^,^13^,^14^,^15^,^22^. Figure 2 (a), (d) and (g) show that for the NM case, two rather flat edge bands derived mainly from the orbitals on the edge atoms cross the Fermi level (*E*_*F*_ = 0), being separated from the bulk projected bands. When the AF is introduced, the two bands split, resulting in an insulating band gap [see Fig. 2 (b), (e) and (h)]. Note that each band is spin (doubly) degenerate. Interestingly, when moving from the AF to FM state, the energy bands of the SiF ZNR remain nearly unchanged except that the two edge bands become fully spin-polarized [Fig. 2 (c)]. In contrast, a Dirac-point like band crossing appears in the band structures of the FM GeF and especially SnF ZNRs [Fig. 2 (f) and (i)]. The band structures of the SnF ZNR in the NM and FM states calculated without the SOC as well as in the FM state with the SOC are displayed in Fig. 3. Figure 3 (left panel) shows that the two edge bands are doubly degenerate in the whole Brillouin zone except near the Γ-point where the inter-edge interaction causes a small splitting. This suggests that the pronounced edge band splitting in Fig. 2(d) and (g) is mainly caused by the stronger SOC in the GeF and SnF ZNRs. A comparison of the middle and right panels in Fig. 3 indicates that the SOC also significantly changes the spin-polarized edge bands in the FM state. In particular, a Dirac-point like band crossing near the Fermi level occurs when the SOC is included (Fig. 3 right panel). Interestingly, the two edge states are on the same edge with the spin-up state moving right and the spin-down state moving left, i.e., they form the helical quantum spin Hall-like edge states. Since the time-reversal symmetry is broken here, the ZNR can be considered as the pseudo-quantum spin Hall insulator^25^. Finally, we notice that as the width of the ZNRs is increased from *n *= 10 to *n *= 17, the calculated energy bands remain more or less unchanged.

As is well known, the relativistic SOC, the coupling between the spin of electron and the fictitious magnetic field created by its own orbital motion around the nucleus, is the fundamental cause of the magnetocrystalline anisotropy energy (MAE). The MAE (*E*_*MA*_) of a magnetic solid is the difference in total electronic energy between two magnetization directions, or the energy required to rotate the magnetization from one direction to another. The MAE determines how strong the magnetization in the solid could be pinned in a given direction and thus is a key property for applications such as permanent magnets and magnetic storage media. Therefore, we have also calculated the MAE for all the considered ZNRs, as listed in Table 3. Here *E*_*MA*_ is defined as the total energy difference between the in-plane and off-plane spin orientations, i.e., *E*_*MA*_ = *E*(in-plane) − *E*(off-plane). A positive *E*_*MA*_ value means that the off-plane spin orientation is preferred. Remarkably, Table 3 shows that the functionalized germanene (GeX) and stanene (SnX) ZNRs possess an extremely large perpendicular magnetic anisotropy, with the *E*_*MA*_ being comparable or even larger than that of the ordered L1_0_FePt alloy (~1.0 meV/f.u.)^26^, which has the largest *E*_*MA*_ among the transition metal alloys. Note that the *E*_*MA*_ of bulk Fe and Ni is in the order of 5.0 μeV/atom^27^. This clearly indicates that the magnetic GeX and SnX ZNRs considered here have promising potentials for use in ultrahigh density magnetic random access memory (MRAM) and other data storage media. Table 3 also indicates that the silicene ZNRs except SiBr and SiI, generally have a much smaller *E*_*MA*_ mainly because of the weak SOC in these systems.

**Figure 4:**
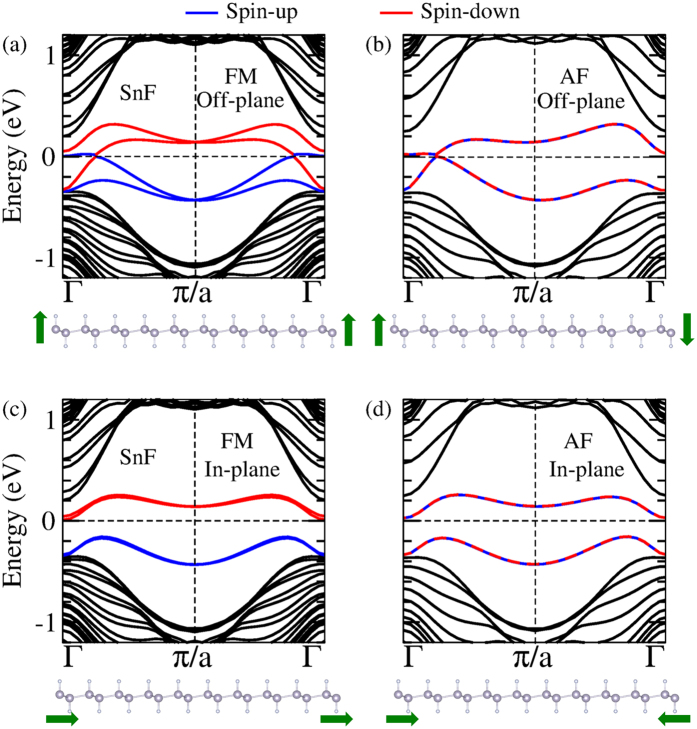
The band structures of the SnF zigzag nanoribbon (ZNR) in the FM (**a,c**) and AF (**b,d**) states. In (**a**) and (**b**), the magnetization is off-plane, and in (**c**) and (**d**), the magnetization is in-plane, as indicated by the green arrows at the bottom of each panel where the side view of the ZNR is also shown. The black curves are bulk bands while the colored curves represent the edge states with the spin orientations indicated by the corresponding colored arrows.

Taking the SOC into account would make the band structure depend on the magnetization direction, and this dependence is the fundamental origin of the MAE discussed above. As an example, we display the band structures of SnF ZNR in the FM and AF states with the off-plane and in-plane magnetizations in Fig. 4. It is clear from Fig. 4 that rotating the magnetization from the off-plane direction to an in-plane direction would change the band structure significantly. Notably, the edge bands would change from the metallic to insulating state. This indicates that the ZNRs can be used as an electric nano-switch operated by rotating magnetization direction. In other words, the ZNRs would exhibit a colossal anisotropic magnetoresistance^28^, and thus would have interesting applications in spintronics. Similar results for the pristine silicene, germanene and stanene ZNRs were obtained from the Kane-Mele-Hubbard Hamiltonian calculations^29^. It is also noted that for the in-plane magnetization, the band structures of the FM and AF states are similar, except that the two edge bands in the FM case are fully spin-polarized, while the two edge bands in the AF case are doubly (spin) degenerate [see Fig. 4 (c) and (d)].

**Figure 5:**
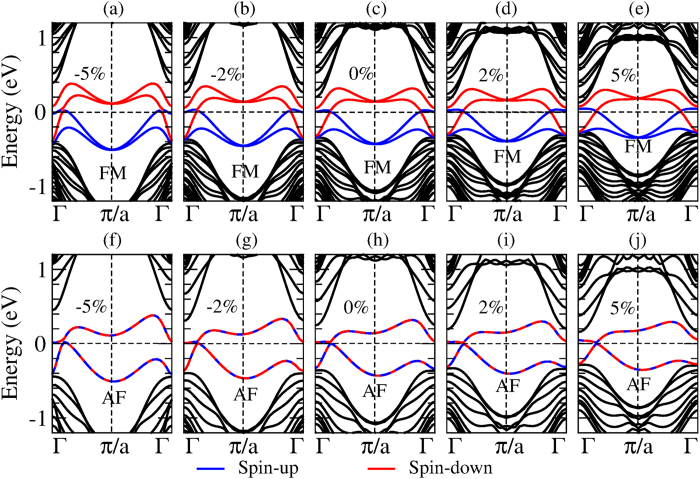
The band structures of the SnF zigzag nanoribbon in the ferromagnetic (FM) (**a–e**) and antiferromagnetic (AF) (**f–j**) configurations under a strain of (**a,f**) −5%, (**b,g**) −2%, (**c,h**) 0%, (**d,i**) 2%, and (**e,j**) 5%. The magnetization is off-plane. The black curves are bulk bands while the blue and red curves represent the spin-up and spin-down states, respectively.

Finally, we consider the effects of an applied strain on the electronic and magnetic properties of the ZNRs. Here we consider the SnX ZNRs as the representatives. Taking the SnF ZNR as an example, Fig. 5 shows the band structures for its off-plane FM and AF configurations under −5%, −2%, 0%, 2% and 5% strains, and Table 4 lists the corresponding electronic and magnetic properties. Clearly, one can tune the electronic and magnetic properties of a ZNR by applying an either compressive or tensile strain. In particular, for the off-plane FM configuration, the band gap is increased monotonically with the tensile strain, while, in contrast, the system becomes metallic below −2% (compressive) strain [see Table 4(a)]. Interestingly, also for the off-plane magnetization, the system prefers the FM state without strain or under a compressive strain, while it becomes the AF state under the tensile strain ≥2% [Table 4 (b)]. Finally, we notice that the exchange coupling strength (*J*) and hence magnetic transition temperature (*T*_*c*_) increases as the strain goes from −5% to 5%.

## Conclusions

We have carried out a systematic study of the magnetic and electronic properties of hydrogenated and halogenated group-IV zigzag nanoribbons (ZNRs) by extensive first-principles density functional calculations. Remarkably, we find that all the functionalized ZNRs have magnetic edge states with rich electronic and magnetic properties tunable by the parent and passivating elements as well as the magnetization direction and external strain. For example, the electric property of the edges can be changed from the conducting to insulating, depending on the parent and passivating elements as well as the applied strain, magnetic configuration and magnetization orientation. The latter controllability is a realization of the colossal anisotropic magnetoresistance for spintronic applications. Moreover, ZNRs such as SiI, Ge, GeI and SnH, have fully spin-polarized metallic edge states and thus are promising materials for spintronics. The calculated MAE can be as large as ~9 meV/edge-site, being 2 × 10^3^ time greater than that of bulk Ni and Fe (~5 μeV/atom), and thus the ZNRs have great potentials for high density magneto-electric data-storage devices. Finally, the calculated exchange coupling strength and thus magnetic transition temperature increases as the applied strain goes from −5% to 5%. Our findings thus show that these ZNRs would have exciting applications in next-generation electronic and spintronic nano-devices.

## Method

The present first-principles calculations are carried out within the generalized gradient approximation (GGA) to the density-functional theory (DFT)^30^,^31^,^32^. The highly accurate projector-augmented-wave (PAW) method^33^ as implemented in the Vienna Ab-Initio Simulation Package (VASP)^34^, is used. We adopt the three-dimensional (3D) supercell approach to model the zigzag nanoribbons. The nanoribbons laid along the *x*-axis are separated by a vacuum space of at least 20 Å along both the *y* and *z* directions. The kinetic energy cutoff for the plane wave expansion used is 400 eV. The total energy criterion for self-consistency in all the electronic structure calculations is set at 10^−6^ eV. A Γ-centered 15 × 15 × 1 Monkhorst-Pack grid^35^ is utilized for the Brillouin zone integration for all the 2D monolayers, while a 50 × 1 × 1 (20 × 1 × 1) grid is adopted in the electronic structure calculations without (with) the SOC for the 1D zigzag nanoribbons.

## Additional Information

**How to cite this article**: Wang, T.-C. *et al*. Tunable magnetic states on the zigzag edges of hydrogenated and halogenated group-IV nanoribbons. *Sci. Rep.* **6**, 39083; doi: 10.1038/srep39083 (2016).

**Publisher's note:** Springer Nature remains neutral with regard to jurisdictional claims in published maps and institutional affiliations.

## Figures and Tables

**Table 1:** Computed topological Z_2_ invariants of hydrogenated (H) and halogenated (F, Cl, Br, I) bulk two-dimensional group IV (Si, Ge, Sn) monolayers.

	Si	Ge	Sn
Pristine	1	1	1
F	0	1	1
Cl	0	1	1
Br	0	1	1
I	0	1	1
H	0	0	0

Z_2_ equals to 1 means topologically nontrivial, and to 0, topologically trivial.

**Table 2:** Calculated lattice constant (*a*_0_), total energy difference between NM and AF configurations (Δ*E*_*NM-AF*_) as well as between the FM and AF configurations (Δ*E*_*FM-AF*_), magnetic moment (*m*_*s*_) (in units of μ_B_ per edge atom), the intra-edge nearest-neighbor exchange coupling parameter (*J*), and estimated mean-field Curie temperature (*T*_*C*_) of hydrogenated (H) and halogenated (F, Cl, Br, I) two-dimensional group IV (Si, Ge, Sn) nanoribbons.

Material	*a*_*0*_(Å)	∆*E*_*NM-AF*_ (meV)	∆*E*_*FM-AF*_ (meV)	*m_s_(μ*_*B*)_	*J* (meV)	*T*_*C*_(K)
Si	3.87	107	0.01	0.61	−0.2	2
SiH	3.86	379	−0.16	1.00	−1.4	11
SiF	3.96	276	0.81	1.01	−1.4	11
SiCl	3.94	237	−0.06	1.01	−5.1	39
SiBr	3.97	204	−0.17	1.02	−5.1	39
SiI	4.06	107	0.56	0.97	−8.3	64
Ge	4.04	43	2.78	0.60	6.2	48
GeH	4.07	204	0.80	1.02	−2.2	17
GeF	4.29	118	−1.42	0.91	13.5	104
GeCl	4.22	72	−1.31	0.88	5.6	43
GeBr	4.24	49	−0.82	0.82	3.9	30
GeI	4.30	8	−0.01	0.46	1.0	8
Sn	4.68	24	0.86	0.52	2.7	21
SnH	4.72	138	0.70	0.98	1.8	14
SnF	5.03	92	−0.12	0.85	17.8	138
SnCl	4.94	77	−0.25	0.84	13.5	104
SnBr	4.92	58	−0.17	0.80	11.1	86
SnI	4.91	30	−0.02	0.68	7.6	59

A positive (negative) *J* value means the FM (AF) exchange coupling between nearest neighboring atoms on the same edge.

**Table 3:** Calculated magnetocrystalline anisotropy energy (*E*_MA_), band gap (*E*_g_), density of states at the Fermi level [*N(E*_*F*_)] (states/eV/unit cell) and its spin-polarization for off-plane and in-plane magnetizations of the hydrogenated (H) and halogenated (F, Cl, Br, I) two-dimensional group IV (Si, Ge, Sn) nanoribbons in the FM state.

Material	*E*_*MA*_(meV)	*E*_*g*_ (meV) (off-plane)	*N(E*_*F*_) (off-plane)	*P(E*_*F*_) (off-plane)	*E*_*g*_ (meV) (in-plane)	*N(E*_*F*_) (in-plane)	*P(E*_*F*_) (in-plane)
Si	0.00	55	0	N/A	55	0	N/A
SiH	−0.01	734	0	N/A	734	0	N/A
SiF	0.00	304	0	N/A	296	0	N/A
SiCl	−0.01	493	0	N/A	492	0	N/A
SiBr	0.13	415	0	N/A	413	0	N/A
SiI	−0.52	0	104.7	−0.99	0	167.1	−1.00
Ge	0.52	0	14.3	−1.00	0	2.1	0.88
GeH	0.59	224	0	N/A	223	0	N/A
GeF	1.01	0	6.0	1.00	11	0	N/A
GeCl	1.01	0	20.0	1.00	6	0	N/A
GeBr	1.39	0	7.8	1.00	55	0	N/A
GeI	−0.18	0	119.7	1.00	0	13.0	0.12
Sn	1.27	0	4.0	0.85	35	0	N/A
SnH	5.28	0	6.5	1.00	114	0	N/A
SnF	8.88	16	0	N/A	169	0	N/A
SnCl	7.52	4	0	N/A	154	0	N/A
SnBr	7.03	24	0	N/A	176	0	N/A
SnI	3.61	12	0	N/A	106	0	N/A

**Table 4:** Calculated band gap (*E*_g_), density of states [*N*(*E*_*F*_)] and spin-polarization *P*(*E*_*F*_) at the Fermi level, intra-edge exchange coupling parameter (*J*) and estimated magnetic transition temperature (*T*_*c*_), spin magnetic moment per edge site (*m*_*s*_), and magnetocrystalline anisotropy energy (*E*_*MA*_) of the ferromagnetic (a) and antiferromagnetic (b) SnF zigzag nanoribbon as a function of strain.

strain	−5%		−2%		0%		2%		5%	
(a) Ferromagnetic case										
*E*_*g*_(off-plane)	0		0		16		18		18	
(meV) (in-plane)	111		145		169		185		204	
*N*(*E*_*F*_) (off-plane)	5.0		7.5		0		0		0	
(states/eV) (in-plane)	0		0		0		0		0	
*P*(*E*_*F*_) (off-plane)	1		1		N/A		N/A		N/A	
(in-plane)	N/A		N/A		N/A		N/A		N/A	
*J* (meV) (off-plane)	10.1		15.6		17.8		19.6		21.5	
*T*_*c*_ (K) (off-plane)	78		122		138		152		167	
*m*_*s*_ (μ_B_) (off-plane)	0.89		0.86		0.85		0.83		0.82	
*E*_*MA*_(meV)	7.10		8.32		8.88		9.20		9.41	
(b) Antiferromagnetic case										
*E*_*g*_(off-plane)		0		7		8		20		15
(meV) (in-plane)		167		183		199		208		220
*N*(*E*_*F*_) (off-plane)		11.7		0		0		0		0
(states/eV) (in-plane)		0		0		0		0		0
*P*(*E*_*F*_) (off-plane)		1		N/A		N/A		N/A		N/A
(in-plane)		N/A		N/A		N/A		N/A		N/A
*ΔE*_FM-AF_(meV) (off-plane)		−1.09		−0.46		−0.12		0.07		0.08
*m*_*s*_ (μ_B_) (off-plane)		.89		0.86		0.85		0.83		0.82
*E*_*MA*_(meV)		7.20		8.35		8.88		9.20		9.41

Note that the *J* and *T*_*c*_ values for the antiferromagnetic nanoribbons are the same as the corresponding ferromagnetic ones.
